# Frailty, walking ability and self-rated health in predicting institutionalization: an 18-year follow-up study among Finnish community-dwelling older people

**DOI:** 10.1007/s40520-020-01551-x

**Published:** 2020-04-18

**Authors:** Anna Viljanen, Marika Salminen, Kerttu Irjala, Päivi Korhonen, Maarit Wuorela, Raimo Isoaho, Sirkka-Liisa Kivelä, Tero Vahlberg, Matti Viitanen, Minna Löppönen, Laura Viikari

**Affiliations:** 1 Health Care Center, Municipality of Lieto, Hyvättyläntie 7, 21420 Lieto, Finland; 2 Faculty of Medicine, Department of Clinical Medicine, Unit of Geriatrics, University of Turku, Turku City Hospital, Kunnallissairaalantie 20, 20700 Turku, Finland; 3 Welfare Division, City of Turku, Yliopistonkatu 30, 20101 Turku, Finland; 4 Faculty of Medicine, Department of Clinical Medicine, Unit of Family Medicine, University of Turku, Turku University Hospital, 20014 Turku, Finland; 5 Faculty of Medicine, Department of Clinical Medicine, Unit of Clinical Chemistry, TYKSLAB, 20521 Turku, Finland; 6 Social and Health Care, City of Vaasa, Ruutikellarintie 4, 65101 Vaasa, Finland; 7 Faculty of Pharmacy, Division of Social Pharmacy, University of Helsinki, 00014 Helsinki, Finland; 8 Institute of Clinical Medicine, Biostatistics, University of Turku, Turku, Finland; 9 Social and Health Care for Elderly, City of Raisio, Sairaalakatu 5, 21200 Raisio, Finland

**Keywords:** Association, Frailty, Institutionalization, Older people, Self-rated health, Walking ability

## Abstract

**Background:**

In clinical practice, there is a need for an instrument to screen older people at risk of institutionalization.

**Aims:**

To analyze the association of frailty, walking-ability and self-rated health (SRH) with institutionalization in Finnish community-dwelling older people.

**Methods:**

In this prospective study with 10- and 18-year follow-ups, frailty was assessed using FRAIL Scale (FS) (*n* = 1087), Frailty Index (FI) (*n* = 1061) and PRISMA-7 (*n* = 1055). Walking ability was assessed as self-reported ability to walk 400 m (*n* = 1101). SRH was assessed by a question of general SRH (*n* = 1105). Cox regression model was used to analyze the association of the explanatory variables with institutionalization.

**Results:**

The mean age of the participants was 73.0 (range 64.0‒97.0) years. Prevalence of institutionalization was 40.8%. In unadjusted models, frailty was associated with a higher risk of institutionalization by FS in 10-year follow-up, and FI in both follow-ups. Associations by FI persisted after age- and gender-adjustments in both follow-ups. By PRISMA-7, frailty predicted a higher risk of institutionalization in both follow-ups. In unadjusted models, inability to walk 400 m predicted a higher risk of institutionalization in both follow-ups and after adjustments in 10-year follow-up. Poor SRH predicted a higher risk of institutionalization in unadjusted models in both follow-ups and after adjustments in 10-year follow-up.

**Discussion:**

Simple self-reported items of walking ability and SRH seemed to be comparable with frailty indexes in predicting institutionalization among community-dwelling older people in 10-year follow-up.

**Conclusions:**

In clinical practice, self-reported walking ability and SRH could be used to screen those at risk.

**Electronic supplementary material:**

The online version of this article (10.1007/s40520-020-01551-x) contains supplementary material, which is available to authorized users.

## Introduction

Frailty in older adults has been described as a phenotype [1] as well as an accumulation of deficits [2, 3]. The prevalence of frailty increases with age [4, 5]. Frailty is more prevalent in women than in men; however, women tolerate frailty better regarding the risk of adverse effects related to frailty [4, 6], such as a higher risk of mortality [5, 7], institutionalization [3, 8, 9] and disability [9, 10].

Frailty is a dynamic state, and the possibility of slowing or reversing the cascade of decline in functional capacity with targeted multicomponent interventions has been proposed [11]. The total healthcare cost increase related to an individual transitioning into a frail state is remarkable, so the possibility of preventing or delaying the transition might lead to substantial cost-savings [12]. Physician’s assessment of a patient’s risk for adverse effects has been shown to be poorer than an objective measurement [13]. A simple measure of an older person’s health in relation to future adverse effects is needed. The recommended [11, 14] screening tools for frailty include the Frail Scale (FS) [15, 16], which is simple to use and can be obtained from data already included in a comprehensive geriatric assessment (CGA) [10, 15]. Also PRISMA-7 is a fast and feasible tool for screening of frailty [17], and has been shown to have high sensitivity and moderate specificity in identifying frailty in community-dwelling older people [18, 19]. The Rockwood Frailty Index is a broadly validated [20] frailty tool with good predictive capability [4, 21].

Slowness is a part of the classic phenotype definition of frailty, the Fried phenotype [1], and often measured as gait speed. Slow gait speed has been found to predict the inability to walk 400 m [22]. Gait speed declines with age and has been shown to predict mortality and institutionalization [23, 24]. Self-reported information on walking ability coincides well with the controlled ability to walk 400 m [25].

Self-rated health (SRH) has been shown to predict mortality [26, 27], institutionalization [28] and future health care expenditure [29] in an elderly population.

We have earlier demonstrated the predictive ability of three frailty tools (Frailty Index, Frail Scale and PRISMA-7) in relation to mortality in Finnish community-dwelling older adults [7]. The aim of the current study was to analyze whether the same frailty tools also predicted a higher risk of institutionalization among the same population during 10- and 18-year follow-ups, and also to analyze the predictive ability of self-reported ability to walk 400 m and self-rated health in association with institutionalization.

## Materials and methods

### Study design and population

This study is a part of the longitudinal epidemiological study carried out in the municipality of Lieto in southwest Finland [30]**.** All persons born in or prior to the year 1933 (*n* = 1596) were invited to participate in the baseline examination that took place between March 1998 and September 1999. Of those eligible, 63 died before they were examined and 273 refused or did not respond leaving 1260 (82%) participants, 533 men and 727 women. An outlier, institutionalized before baseline at the age of 17, was excluded from the analyses leaving 1259 participants.

At baseline the study protocol consisted of an extensive interview on demographic and socioeconomic factors and health behavior, numerous laboratory tests, and a clinical examination including a comprehensive survey of the participants’ medical records.

The follow-ups time were 10 and 18 years. Participants no longer living in Lieto at the end of 2016 (*n* = 86) were excluded from the present analyses predicting institutionalization, as it was not possible to ascertain whether they continued living at home or were institutionalized in another municipality. Also participants already living in institutional care (*n* = 67) at baseline were excluded from the institutionalization analyses. This left us with 1106 participants.

### Mortality

Data from all participants who died before January 2017 were obtained from the official Finnish Cause of Death Registry using unique personal identification numbers.

### Institutionalization

A total of 395 participants were institutionalized. Institutionalization was defined as permanent entry into a nursing home of which the data were gathered from the municipality’s electronic patient record system and coded by month and year of entry.

### Frailty

Frailty was characterized using three commonly used approaches: FRAIL scale (FS) [15, 16], Frailty Index (FI) [2, 3] and PRISMA-7 [31]. The information on participants’ frailty status were gathered from baseline data.

The FS is a frailty screening tool based on the Fried frailty phenotype [1]. The original phenotype has been modified in several studies and has not lost its prognostic significance [32]. The FS uses five self-reported items [15, 16]. We used a slightly modified version of FS (Appendix 1). In addition, data of illnesses were gathered from patient records instead of self-reporting.

The FI is calculated as the proportion of individual’s deficits in relation to the total amount of deficits chosen [2, 3]. In this study we used an FI consisting of 36 deficits (Appendix 2). We used pre-described cut-points of FI ≤ 0.08 for robust, FI 0.09‒0.24 for pre-frail and FI ≥ 0.25 for frail [4].

PRISMA-7 includes seven self-reported items [31], and in this study we used a modified version (Appendix 3).

### Walking ability

Walking ability was assessed by self-reported ability to walk 400 m independently (yes/no).

### Self-rated health

The information about the participants’ SRH was gathered at baseline by the question: “How would you describe your current state of health?” The answers were categorized into three groups: ‘good’, ‘intermediate’ and ‘poor’.

### Ethics

The study was conducted according to the guidelines of the Declaration of Helsinki. The Ethics Committee of the Hospital District of Southwest Finland approved the study protocol. Participants provided written informed consent for the study.

### Statistical analyses

At baseline, differences between men and women were tested using the Chi squared test, Fisher’s exact test or two-sample *t* test.

Hazard ratios (HRs) and their 95% confidence intervals for institutionalization were calculated using Cox proportional hazard models. Proportional hazards assumption was tested using Martingale residuals. The follow-up periods were calculated from the baseline measurements to the end of the follow period of 10 and 18 years or to the death of the individual. We used death as a competitive factor in the analyses.

Firstly, unadjusted Cox regression analyzes were conducted for frailty tools, self-reported walking ability and SRH. Secondly, Cox regression analyzes for FI, FS, self-reported walking ability and SRH were adjusted for age and gender, which are items of PRISMA-7. The interaction between gender and all explanatory variables (frailty tools, walking ability and SRH) were included in Cox regression model. *P* values less than 0.05 were considered statistically significant. All statistical analyzes were performed using SAS System for Windows, version 9.4 (SAS Institute Inc., Cary, NC, USA).

## Results

### Baseline characteristics

The mean age of the participants was 73.0 (SD 6.4, range 64.0‒97.0) years and 57% were women. More detailed baseline characteristics of 1106 study participants are shown in Table 1.

**Table 1:** Baseline characteristics of study participants (n = 1106)

	*n* (%)
Age, mean (SD), range	73.0 (6.4), 64.0–97.0
Age	
64–74	720 (65)
75–84	312 (28)
≥ 85	74 (7)
Women	633 (57)
Living alone	342 (31)
Education	
More than basic^a^ or basic	94 (8)
Less than basic	1012 (92)
MMSE < 26	270 (24)
Body mass index, kg/m^2^	
< 20	42 (4)
20–24.9	297 (27)
25–29.9	495 (45)
30–34.9	208 (19)
≥ 35	61 (6)
Number of prescribed medicines	
< 5	833 (75)
5–7	196 (18)
8–9	54 (5)
≥ 10	23 (2)

^a^Six years of elementary school

**Fig. 1:**
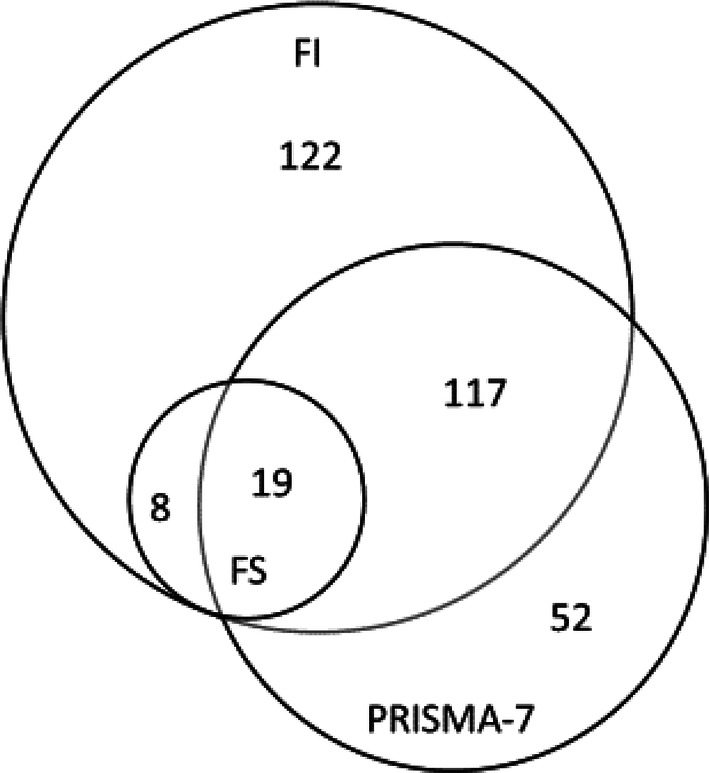
Venn diagram of overlapping participants categorized as frail by FS (*n* = 27), FI (*n* = 266) and PRISMA-7 (*n* = 188)

Three percent of participants were characterized as frail according to FS, 25% according to FI and 18% according to PRISMA-7. Figure 1 shows a Venn diagram of overlapping participants categorized as frail by FS, FI and PRISMA-7. All of the participants categorized as frail by FS were also categorized frail by FI. Frailty (both pre-frailty and frailty) was more common in women than in men according to FI and FS, but according to PRISMA-7, more men were frail than women.

Only 8% (9% of women and 7% of men) of the participants were self-reportedly unable to walk 400 m.

Altogether 14% (*n* = 159) of participants rated their health as poor. The proportions of men and women in different groups of SRH (good, intermediate and poor) were similar: 40, 46 and 15% for men, and 41, 45 and 14% for women, respectively.

### Follow-up characteristics

Of the participants, 212 (31.1%) women and 130 (26.5%) men were alive at the end of 2016.

Of the participants self-reportedly able to walk 400 m at baseline, 65% were still living at home, 12% institutionalized and 23% deceased after 10 years (at the end of 2008). After 18 years (at the end of 2016) the proportions were 28, 28 and 44%, respectively. Of the participants self-reportedly unable to walk 400 m, 10% were living at home, 43% institutionalized and 47% deceased after 10 years. After 18 years the proportions were 0, 47 and 53%, respectively.

Of the participants with good SRH at baseline, 72% were still living at home, 12% institutionalized and 16% deceased after 10 years. After 18 years, the proportions were 32, 29 and 39%, respectively. Of the participants with poor SRH, 29% were living at home, 26% institutionalized and 45% deceased after 10 years. After 18 years, the proportions were 6, 40 and 53%, respectively.

### Prevalence of institutionalization

When analyzing the proportion of participants institutionalized in the municipality of Lieto, we included participants who had been institutionalized before baseline. We only took into account the participants deceased by the end of 2016, to count the proportion of participants institutionalized during their lifetime. This left us with 831 participants, of which 339 were institutionalized (40.8%). There was a higher prevalence of institutionalization in women (48.9%) than in men (30.2%).

The mean age of the participants at the time of institutionalization and the mean time spent living in an institution are shown in Table 2.

**Table 2:** Age of study participants at time of institutionalization and mean time spent living in an institution

	Mean age in years at time of institutionalization (SD)				Mean time in years spent living in an institution (SD)			
	Before baseline	Baseline–January 2008	January 2008–January 2017	Study population	Before baseline	Baseline–January 2008	January 2008–January 2017	Study population
Both [*n*]	81.6 (7.0) [67]	83.9 (5.9) [160]	87.4 (5.3) [112]	84.6 (6.3) [339]	5.2 (3.8)	3.2 (2.7)	1.7 (1.5)	3.1 (2.9)
Men [*n*]	81.1 (6.6) [18]	82.3 (5.6) [54]	85.4 (5.0) [37]	83.1 (5.8) [109]	4.5 (3.6)	2.7 (2.6)	1.5 (1.4)	2.6 (2.7)
Women [*n*]	81.8 (7.1) [49]	84.7 (5.9) [106]	88.4 (5.1) [75]	85.3 (6.4) [230]	5.4 (3.9)	3.5 (2.8)	1.9 (1.5)	3.4 (3.3)

### Cox models for frailty and institutionalization

During the 10-year follow-up, both being frail and pre-frail according to FS and FI were associated with a higher risk of institutionalization in unadjusted Cox regression models (Table 3). After age- and gender-adjusted (items of PRISMA-7) analyses for FS and FI, the associations persisted for FI; pre-frailty according to FS also remained significantly related to higher risk of institutionalization. Also using the binary (robust or frail) PRISMA-7, being frail was associated with a higher risk of institutionalization during the 10-year follow-up.

**Table 3:** Unadjusted and adjusted hazard ratios (HR) and their 95% confidence intervals (CI) (in parentheses) of frailty indexes, self-reported ability to walk 400 m and self-rated health for institutionalization during the 10-year follow-up

	Non-institutionalized *n* (%)	Institutionalized *n* (%)	Deceased *n* (%)	Unadjusted HR (95% CI)	*P*-value	Adjusted R (95% CI)^a^	*P*-value
FRAIL scale (*n* = 1087)							
Robust (*n* = 710)	496 (70)	74 (10)	140 (20)	1		1	
Pre-frail (*n* = 350)	158 (45)	75 (21)	117 (33)	**2.25 (1.63–3.10)**	** < .001**	**1.48 (1.04–2.10)**	**.030**
Frail (*n* = 27)	1 (4)	8 (30)	18 (67)	**3.32 (1.57–7.00)**	**.002**	1.33 (0.57–3.11)	.505
Frailty index (*n* = 1061)							
Robust (*n* = 197)	164 (83)	8 (4)	25 (13)	1		1	
Pre-frail (*n* = 596)	402 (67)	64 (11)	130 (22)	**2.74 (1.32–5.69)**	**.007**	**2.32 (1.12–4.79)**	**.023**
Frail (*n* = 268)	76 (28)	79 (29)	113 (42)	**8.82 (4.28–18.20)**	** < .001**	**4.50 (2.11–9.63)**	** < .001**
PRISMA-7 (*n* = 1055)							
Robust (*n* = 860)	613 (71)	85 (10)	162 (19)	1			
Frail (*n* = 195)	42 (22)	63 (32)	90 (46)	**3.95 (2.85–5.49)**	** < .001**		
Self-reported ability to walk 400 m (*n* = 1101)							
Yes (*n* = 1011)	653 (65)	123 (12)	235 (23)	1		1	
No (*n* = 90)	9 (10)	39 (43)	42 (47)	**4.82 (3.29–7.05)**	** < .001**	**2.06 (1.25–3.41)**	**.005**
Self-rated health (*n* = 1105)							
Good (*n* = 446)	321 (72)	52 (12)	73 (16)	1		1	
Moderate (*n* = 500)	298 (60)	68 (14)	134 (27)	1.18 (0.82‒1.69)	.380	1.07 (0.74‒1.54)	.73
Poor (*n* = 159)	46 (29)	42 (26)	71 (45)	**2.51 (1.67‒3.77)**	** < .0001**	**1.59 (1.00‒2.53)**	**.05**

^a^Values are adjusted for age and gender (items included in PRISMA-7)

**Fig. 2:**
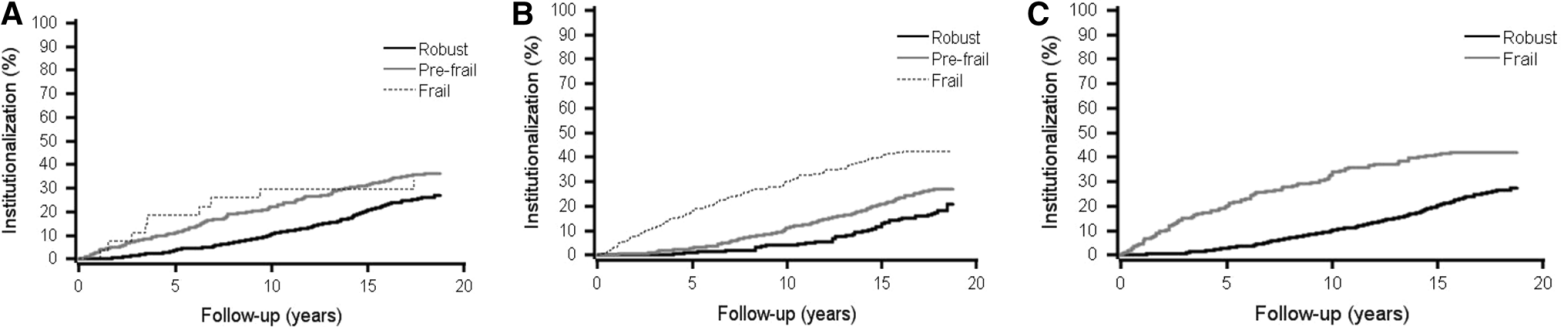
Rates of institutionalization with death as a competing risk by Frail Scale (**a**), Frailty Index (**b**), and PRISMA7 (**c**) during the 18-year follow-up

During the 18-year follow-up, being pre-frail according to FS and both being pre-frail and frail according to FI were associated with a higher risk of institutionalization in unadjusted models (Table 4). After adjustments for age and gender, only being frail according to FI significantly associated with a higher risk of institutionalization. Also being frail according to PRISMA-7, was significantly related to a higher risk of institutionalization. Figure 2 shows the rates of institutionalization by FI, FS and PRISMA-7 during the 18-year follow-up.

**Table 4:** Unadjusted and adjusted hazard ratios (HR) and their 95% confidence intervals (CI) (in parentheses) of frailty indexes, self-reported ability to walk 400 m and self-rated health for institutionalization during the 18-year follow-up

	Non-institutionalized *n* (%)	Institutionalized *n* (%)	Deceased *n* (%)	Unadjusted HR (95% CI)	*P*-value	Adjusted^a^ HR (95% CI)	*P*-value
FRAIL scale (*n* = 1087)							
Robust (*n* = 710)	221 (31)	185 (26)	304 (43)	1		1	
Pre-frail (*n* = 350)	61 (17)	126 (36)	163 (47)	**1.56 (1.24–1.96)**	** < .001**	1.22 (0.96–1.55)	.110
Frail (*n* = 27)	0 (0)	9 (33)	18 (67)	1.50 (0.72–3.09)	.278	0.82 (0.37–1.81)	.629
Frailty index (*n* = 1061)							
Robust (*n* = 197)	73 (37)	36 (18)	88 (45)	1		1	
Pre-frail (*n* = 596)	192 (32)	159 (27)	245 (41)	**1.56 (1.10–2.21)**	**.013**	1.38 (0.98–1.97)	.068
Frail (*n* = 268)	11 (4)	114 (43)	143 (53)	**3.08 (2.13–4.46)**	** < .001**	**2.00 (1.34–2.97)**	** < .001**
PRISMA-7 (*n* = 1055)							
Robust (*n* = 860)	278 (32)	228 (27)	354 (41)	1			
Frail (*n* = 195)	6 (3)	82 (42)	107 (55)	**2.03 (1.55–2.66)**	** < .001**		
Self-reported ability to walk 400 m (*n* = 1101)							
Yes (*n* = 1011)	284 (28)	285 (28)	442 (44)	1		1	
No (*n* = 90)	0 (0)	42 (47)	48 (53)	**2.31 (1.59–3.36)**	** < .001**	1.28 (0.82–2.01)	.305
Self-rated health (*n* = 1105)							
Good (*n* = 446)	143 (32)	128 (29)	175 (39)	1		1	
Moderate (*n* = 500)	133 (27)	136 (27)	231 (46)	0.96 (0.76‒1.22)	.75	0.91 (0.72‒1.16)	.461
Poor (*n *= 159)	10 (6)	64 (40)	85 (53)	**1.65 (1.21‒2.25)**	**.002**	1.29 (0.92‒1.81)	.137

^a^Values are adjusted for age and gender (items included in PRISMA-7)

The association of frailty and institutionalization did not significantly differ between men and women either in 10- or 18-year follow-up by FI or PRISMA-7; using FS, being pre-frail predicted a significantly higher risk of institutionalization in women (1.85 [1.41–2.43], *p* < 0.001), but not in men (0.98 [0.63‒1.53], *p* = 0.930) during the 18-year follow-up.

### Cox models for walking ability, self-rated health and institutionalization

**Fig. 3:**
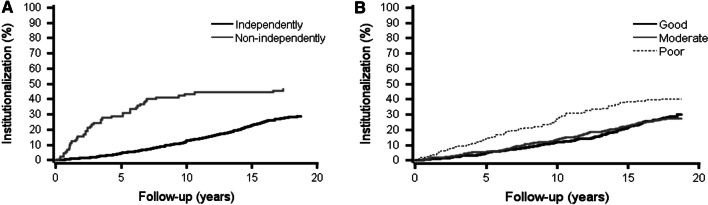
Rates of institutionalization with death as a competing risk by self-reported ability to walk 400 m (**a**) and self-rated health (**b**) during the 18-year follow-up

In an unadjusted model, the self-reported inability to walk 400 m and poor SRH were associated with a higher risk of institutionalization during both follow-ups. After adjustments, the associations persisted in 10-year follow-up. Figure 3 shows rates of institutionalization by self-reported walking ability and SRH during the 18-year follow-up.

No significant interaction was found between gender and self-reported walking ability in predicting the risk of institutionalization during either follow-ups. Poor SRH, instead, predicted a higher risk of institutionalization in women (1.85 [1.27‒2.68], *p* = 0.001) but not in men (1.49 [0.86‒2.60], *p* = 0.157) during the 18-year follow-up.

## Discussion

In our study, the prevalence of frailty varied from 3% (according to FS) to 25% (according to FI). Being frail according to FI and PRISMA-7 was associated with a higher risk of institutionalization in both follow-ups. Also being pre-frail according to FS was associated with a higher risk of institutionalization in 10-year follow-up. The self-reported inability to walk 400 m and poor SRH were associated with a higher risk of institutionalization in 10-year follow-up.

The current estimated prevalence of frailty in European adults aged 65 years and older is 10‒20% [33], but the estimate varies between population samples and the frailty tools used [4, 33]. Our findings were similar to previous studies regarding gender differences in categorizing participants as frail [6]. The current study also supports results of earlier studies showing that frailty tools differ in how they classify participants as frail and how they do not capture the same individuals, but still are capable of predicting adverse effects of frailty [9, 19].

Being frail according to FI was associated with a higher risk of institutionalization during both 10 and 18 year of follow-up also in adjusted analyses. Because of low number of subjects categorized as frail, only being pre-frail according to FS was related to higher risk of institutionalization during the 10-year follow-up. Frailty according to PRISMA-7 predicted a higher risk of institutionalization in both follow-ups. These results are in consistence with previous research [4, 8]. In our previous study, we have also demonstrated that frailty according to all these three frailty tools predicted mortality up to 18 years [7].

In our study, the self-reported inability to walk 400 m at baseline was associated with a higher risk of institutionalization in 10-year follow-up. This is similar to previous research regarding walking ability [34]. In fact, the predictive ability of slow gait speed in regarding mortality and institutionalization has been found comparable to the FI [35]. Also similar to earlier research [28], we found that poor SRH at baseline was associated with a higher risk of institutionalization in 10-year follow-up. The finding is notable, since information about SRH is easy to acquire, with the exception of individuals with cognitive decline, whose assessment of their own health is not reliable due to loss of insight [36]. Poor SRH predicted a higher risk of institutionalization in women but not in men during the 18-year follow-up. Gender differences have been found earlier, but not always in the same direction [26, 27].

We also found that the mean age at the time of institutionalization has risen during the follow-up-period and the mean time spent living in an institution decreased. Women were institutionalized at a higher age than men and lived in an institution longer than men, reflecting that women seem to tolerate frailty and functional disabilities better than men [4, 6]. Besides living longer, older people are staying longer at home than in previous years. These findings reflect the rising of the life expectancy of the Finnish older people during the follow-up period [37], and possibly also the change in the municipality’s policy of the elderly care in which home care is preferred for many reasons, of which one is the high cost of institutional care. More women were institutionalized than men, due to the fact that women live longer, and perhaps do not have informal care available to them as often as to men. The role of formal or informal care at home, possibly affecting institutionalization, was not addressed in this study.

The strengths of our study are the large sample size and a long follow-up period enabling broad generalizability to the community-dwelling older population. We also used death as a competitive factor in our analyses. The frailty tools used were validated and commonly used, although the modified versions of FS and PRISMA-7 could have had an impact on our results. Especially, prevalence of frailty was low according to FS, and it’s possible that the modifications used in our study underestimated the prevalence, and thus the association with institutionalization. The most obvious limitation to our study was that all studied explanatory variables were only assessed at baseline.

To conclude, a simple self-reported item, such as self-reported ability to walk 400 m and/or self-rated health could be used in the clinical setting instead of more time-consuming frailty tools, to screen for future risk of institutionalization, and identify older persons in need of a comprehensive evaluation and possible interventions.

## Electronic supplementary material

Below is the link to the electronic supplementary material.

Supplementary file1 (DOCX 23 kb)

Supplementary file2 (DOCX 26 kb)

Supplementary file3 (DOCX 23 kb)
