# Supplementary material for: Frailty, walking ability and self-rated health in predicting institutionalization: an 18-year follow-up study among Finnish community-dwelling older people
Source: Aging Clin Exp Res. 2020 Apr 18;33(3):547–54. doi: 10.1007/s40520-020-01551-x (PMC7943499; doi:10.1007/s40520-020-01551-x)
Supplement: Supplementary file 1 — Supplementary file1 (DOCX 23 kb) [file 40520_2020_1551_MOESM1_ESM.docx]

**Appendix 1** Modified FRAIL scale in total population and by gender

| FRAIL scale items | Total population  (n = 1087)  n (%) | Women  (n = 624)  n (%) | Men  (n = 463)  n (%) | P-value^a^ |
| --- | --- | --- | --- | --- |
| Getting tired for no reason | 247 (23) | 158 (25) | 89 (19) | .002 |
| Inability to climb stairs | 75 (7) | 57 (9) | 18 (4) | .001 |
| Inability to walk 400 m | 87 (8) | 57 (9) | 30 (6) | .111 |
| At least 5 illnesses^b^ | 3 (0) | 2 (0) | 1 (0) | .745 |
| Losing weight some of the time–most of the time | 110 (10) | 63 (10) | 47 (10) | .976 |
|  |  |  |  |  |
| FRAIL scale |  |  |  | .015 |
| Robust (0 points) | 710 (65) | 386 (62) | 324 (70) |  |
| Pre-frail (1–2 points) | 350 (32) | 219 (35) | 131 (28) |  |
| Frail (3–5 points) | 27 (2) | 19 (3) | 8 (2) |  |

^a^P-value for the differences between genders

^b^High blood pressure, diabetes, cancer, chronic lung disease, myocardial infarction, congestive heart failure, angina, asthma, arthritis, stroke, and kidney disease
