# Supplementary material for: Frailty, walking ability and self-rated health in predicting institutionalization: an 18-year follow-up study among Finnish community-dwelling older people
Source: Aging Clin Exp Res. 2020 Apr 18;33(3):547–54. doi: 10.1007/s40520-020-01551-x (PMC7943499; doi:10.1007/s40520-020-01551-x)
Supplement: Supplementary file 2 — Supplementary file2 (DOCX 26 kb) [file 40520_2020_1551_MOESM2_ESM.docx]

**Appendix 2** Frailty index in total population and by gender

| Frailty index items | Total population  (n = 1061)  n (%) | Women  (n = 604)  n (%) | Men  (n = 457)  n (%) | P-value^a^ |
| --- | --- | --- | --- | --- |
| Needs help with toileting | 7 (1) | 4 (1) | 3 (1) | .987 |
| Needs help with dressing and undressing | 25 (2) | 9 (1) | 16 (4) | .032 |
| Needs help with preparing meals | 118 (11) | 49 (8) | 69 (15) | <.001 |
| Needs help with house work | 136 (13) | 72 (12) | 64 (14) | .313 |
| Needs help with heavy household chores | 356 (34) | 236 (39) | 120 (26) | <.001 |
| Needs help with personal care | 70 (7) | 45 (7) | 25 (5) | .199 |
| Needs help with moving about inside house | 41 (4) | 31 (5) | 10 (2) | .014 |
| Arthritis or rheumatism | 72 (7) | 36 (6) | 36 (8) | .219 |
| High blood pressure | 378 (36) | 229 (38) | 149 (33) | .007 |
| Chronic bronchitis or emphysema | 38 (4) | 11 (2) | 27 (6) | <.001 |
| Diabetes mellitus | 125 (12) | 66 (11) | 59 (13) | .321 |
| Heart disease^b^ | 325 (31) | 170 (28) | 155 (34) | .043 |
| Cancer | 100 (9) | 58 (10) | 42 (9) | .820 |
| Stomach or intestinal ulcers | 58 (5) | 28 (5) | 30 (7) | .171 |
| Suffers from the effect of stroke | 44 (4) | 16 (3) | 28 (6) | .005 |
| Urinary incontinence | 205 (20) | 181 (30) | 24 (5) | <.001 |
| Stool incontinence | 5 (0) | 5 (1) | 0 (0) | .051 |
| Hip or femoral fracture | 6 (1) | 4 (1) | 2 (0) | .629 |
| Shortness of breath | 687 (67) | 403 (69) | 284 (64) | .076 |
| Angina pectoris | 491 (46) | 265 (44) | 226 (49) | .079 |
| Other medical problems^c^ | 622 (59) | 374 (62) | 248 (54) | .012 |
| No regular physical exercise | 279 (27) | 170 (29) | 109 (24) | .115 |
| Vision problem | 45 (4) | 32 (5) | 13 (3) | .049 |
| Hearing problem | 47 (4) | 15 (2) | 32 (7) | <.001 |
| Feeling hopeless | 172 (16) | 109 (18) | 63 (14) | .064 |
| Emotional problem | 75 (7) | 53 (9) | 22 (5) | .013 |
| Memory problem | 88 (8) | 44 (7) | 44 (10) | .151 |
| Bodily pain | 498 (47) | 302 (50) | 196 (43) | .022 |
| Speech problem | 5 (1) | 0 (0) | 5 (2) | .032 |
| Resting tremor | 35 (3) | 21 (4) | 14 (3) | .709 |
| Five or more medications | 376 (35) | 232 (38) | 144 (32) | .020 |
| Difficulties carrying or lifting light loads | 435 (41) | 308 (51) | 127 (28) | <.001 |
| Mobility problem | 173 (16) | 113 (19) | 60 (13) | .015 |
| Limited kind of amount of activity | 89 (8) | 54 (9) | 35 (8) | .456 |
| Feeling tired all the time | 31 (3) | 21 (3) | 10 (2) | .217 |
| Weight loss | 502 (47) | 282 (47) | 220 (48) | .639 |
|  |  |  |  |  |
| Frailty index |  |  |  | <.001 |
| Robust (≤0.08) | 197 (19) | 87 (14) | 110 (24) |  |
| Pre-frail (0.09–0.24) | 596 (56) | 344 (57) | 252 (55) |  |
| Frail (≥0.25) | 268 (25) | 173 (28) | 95 (21) |  |

^a^P-value for the differences between genders

^b^Known heart disease at baseline (ICD-10: I20-I25, I48, I49)

^c^Other disease at baseline (ICD-10: E03, E05, G20, G35, J44-J46, M15-M17, M47)
