# Supplementary material for: Frailty, walking ability and self-rated health in predicting institutionalization: an 18-year follow-up study among Finnish community-dwelling older people
Source: Aging Clin Exp Res. 2020 Apr 18;33(3):547–54. doi: 10.1007/s40520-020-01551-x (PMC7943499; doi:10.1007/s40520-020-01551-x)
Supplement: Supplementary file 3 — Supplementary file3 (DOCX 23 kb) [file 40520_2020_1551_MOESM3_ESM.docx]

**Appendix 3** Modified PRISMA-7 in total population and by gender

| PRISMA-7 items | Total population  (n = 1055)  n (%) | Women  (n = 599)  n (%) | Men  (n = 456)  n (%) | P-value^a^ |
| --- | --- | --- | --- | --- |
| Age > 85 years | 45 (4) | 29 (5) | 16 (4) | .288 |
| Man | 456 (43) | 0 (0) | 456 (100) |  |
| Rather poor or poor self-rated health | 143 (14) | 76 (13) | 67 (15) | .346 |
| Needs help with taking care of personal hygiene and/or with taking care of finances | 130 (12) | 88 (14) | 45 (10) | .034 |
| Inability to move outdoors | 37 (4) | 28 (5) | 9 (2) | .018 |
| Having someone close to count on in a case of need for help | 974 (92) | 557 (93) | 417 (91) | .352 |
| Use of a stick, walker or wheelchair | 163 (15) | 109 (18) | 54 (12) | .005 |
|  |  |  |  |  |
| PRISMA-7 |  |  |  | <.001 |
| Robust (0–2 points) | 860 (82) | 519 (87) | 341 (75) |  |
| Frail (≥3 points) | 195 (18) | 80 (13) | 115 (25) |  |

^a^P-value for the differences between genders
